# Incidence and risk factors of hypotension in cesarean section patients under spinal anesthesia at a referral hospital in Mogadishu, Somalia

**DOI:** 10.1186/s12884-025-08024-x

**Published:** 2025-08-25

**Authors:** Asha Abdullahi Barud, Ikran Abdulkadir Ali, Nasra Mohamud Hilowle, Hiba Bashir Hassan, Iftin Mohamed Osman

**Affiliations:** 1 Department of Anesthesia, Mogadishu Somalia Turkey Recep Tayyip Erdoğan Training and Research Hospital, Mogadishu, Somalia; 2 Department of Neonatal Intensive Care Unit, Yardimeli Hospital, Mogadishu, Somalia; 3 Department of Obstetrics and Gynecology, Mogadishu Somalia Turkey Recep Tayyip Erdoğan Training and Research Hospital, Mogadishu, Somalia; 4 Education Department, Mogadishu Somalia Turkey Recep Tayyip Erdoğan Training and Research Hospital, Mogadishu, Somalia

**Keywords:** Incidence, Associated risk factors, Hypotension, Cesarean section, Spinal anesthesia

## Abstract

**Background:**

Spinal anesthesia-induced hypotension is a common complication during cesarean sections, with significant implications for maternal and neonatal outcomes. However, data on the incidence and risk factors of hypotension in Africa, notably in Somalia, are limited. This study aimed to investigate the incidence and risk factors of hypotension in cesarean section patients under spinal anesthesia at a referral hospital in Mogadishu, Somalia.

**Methods:**

A cross-sectional study was conducted at a referral hospital in Mogadishu, Somalia, involving 320 patients who underwent cesarean section under spinal anesthesia using a systematic random sampling technique. Data on sociodemographic, medical history, and anesthetic characteristics were collected using a questionnaire. Descriptive statistics were used for frequencies and percentages. Chi-square tests and logistic regression were used to identify the risk factors associated with hypotension after spinal anesthesia at α = 0.05.

**Results:**

Among the 320 participants, the incidence of hypotension after spinal anesthesia during cesarean section was 78.1% (95%CI = 73.8–82.5]. In the multivariable model, five variables were found to be significantly associated with hypotension after spinal anesthesia in patients undergoing cesarean section: Mothers with BMI above 25 kg/m^2^ (AOR = 2.65, 95% CI = 1.39–5.04), mothers who had no hypertension during pregnancy (AOR = 6.00, 95% CI = 2.50–14.40), mothers who did not take preload IV fluid (AOR = 3.06, 95% CI = 1.22–7.66), mothers who did not take ephedrine medication (AOR = 8.84, 95% CI = 4.10-19.05), patients below 11 g/dl of preoperative hemoglobin (AOR = 2.52, 95% CI = 1.25–5.05).

**Conclusions:**

This study found a high incidence of hypotension among mothers undergoing cesarean section under spinal anesthesia in Mogadishu. Significant risk factors included high BMI, low preoperative hemoglobin, lack of preload and absence of ephedrine use. Targeted interventions—such as preoperative risk screening, hemoglobin optimization, and prophylactic ephedrine use—are recommended to reduce the occurrence of spinal anesthesia-induced hypotension.

## Background

Spinal anesthesia, also known as subarachnoid block, is a widely used regional anesthetic technique in obstetric surgery, particularly during cesarean sections. It involves the injection of local anesthetics into the subarachnoid space, resulting in rapid sensory and motor blockade below the level of administration. Compared to general anesthesia, spinal anesthesia is considered safer due to its simplicity, lower maternal mortality rate, and avoidance of airway manipulation. Despite these advantages, spinal anesthesia is associated with several complications, with spinal anesthesia-induced hypotension (SAIH) being the most common and clinically significant complication. Other complications include bradycardia, post-dural puncture headache, nausea, and vomiting. Among these, hypotension remains the most frequent and concerning adverse effect during cesarean delivery under spinal anesthesia [1–4].

The magnitude of SAIH varies significantly across different settings. Studies report that hypotension can occur in 25–75% of pregnant women undergoing cesarean section under spinal anesthesia, depending on the diagnostic criteria used [5–8]. For instance, an incidence of up to 71% has been documented, making this a major clinical challenge. In South Africa, spinal hypotension accounts for more than half of the 2% of maternal deaths attributed to anesthesia-related complications [9]. In Ethiopia, 68.2% of cesarean sections are performed under spinal anesthesia, with a reported complication rate consistent with global trends [4]. These findings underscore the high burden of hypotension during spinal anesthesia, particularly in low-resource settings.

The pathophysiological mechanism of SAIH is largely attributed to the sympathetic blockade caused by local anesthetics. This leads to vasodilation, decreased venous return, and reduced cardiac output. In pregnant women, physiological changes such as compression of the inferior vena cava by the gravid uterus and increased circulating blood volume further exacerbate the risk of hypotension. Clinically, spinal hypotension can result in nausea, vomiting, dizziness, loss of consciousness, fetal acidosis, cardiac arrest, and even maternal or fetal death in severe cases [10, 11]. The maternal-fetal implications of SAIH necessitate effective prevention and management strategies.

Several risk factors are associated with SAIH. These include advanced maternal age, which alters cardiac reserve and baroreceptor sensitivity; high body mass index (BMI), which increases intra-abdominal pressure and reduces cerebrospinal fluid volume; and other physiological alterations such as reduced vascular tone and increased venous capacitance [12, 13]. Various interventions have been proposed to mitigate the risk of hypotension, including preloading or coloading with intravenous fluids, the use of vasopressors (e.g., ephedrine or phenylephrine), left uterine displacement, and leg wrapping. Despite these strategies, the incidence rate remains high, indicating the need for context-specific preventive approaches.

Although SAIH has been studied extensively in various regions, there is a notable lack of data from Somalia, particularly in Mogadishu. The absence of recent, locally relevant studies limits evidence-based practice in anesthesia management. Given the high incidence of cesarean sections performed under spinal anesthesia in Mogadishu and the observed complications, there is an urgent need for further research. This study sought to address this gap by investigating the incidence and risk factors of hypotension in patients undergoing cesarean section under spinal anesthesia at a referral hospital in Mogadishu, Somalia. The findings from this research are expected to guide clinical practice, improve maternal and fetal outcomes, and inform future studies and national guidelines in similar resource-constrained settings.

## Methods and materials

### Study design and study setting

A cross-sectional study was conducted to examine the incidence and risk factors associated with hypotension in patients undergoing cesarean section under spinal anesthesia at a referral hospital in Mogadishu, Somalia from May1 to July 30, 2024. This study was conducted in Mogadishu, the capital city of Somalia, with nearly 3.5 million population living in this city, 20 districts, and more than 30 hospitals, including public and private hospitals. The Mogadishu Somalia Turkey Recep Tayyip Erdoğan Training and Research Hospital is the largest medical center in the city. It receives patients from hospitals various cities. This hospital was chosen for the study because it handles many childbirth cases and has skilled anesthetists. The hospital has eight operating rooms, twelve delivery rooms, thirty adult ICU beds, six pediatric ICU beds, twenty NICU incubators, and over two hundred emergency and inpatient beds. The staff includes 1,500 people, including managers, healthcare workers, technicians, cleaners, and security staff.

### Study population

Pregnant mothers who attended the operation theatre for cesarean section at a referral hospital were included in this study.

### Eligibility criteria

Pregnant mothers who underwent cesarean section under spinal anesthesia and voluntarily participated in the study were included. Patients in whom spinal anesthesia was converted to general anesthesia and those with contraindications to spinal anesthesia were excluded from the study.

### Sample size estimation and sampling techniques

The required sample size was determined using the standard cross-sectional study formula [14]: *n* = [Z_2/α_^2^ P(1-P)]/e^2^, where n represents the necessary sample size, Z is the standard normal distribution value for the desired confidence level (Z = 1.96, 95% CI), P is the anticipated true proportion, and e is the desired precision. Assuming *p* = 0.746 [15] and e = 0.05, a sample size of 291 was calculated. An additional 10% was added to account for attrition, resulting in a final sample size of 320 participants. The study utilized a systematic random sampling approach to select participants, with an interval between every two patients, taking one of the daily pregnant patients admitted to the hospital for cesarean section.

### Research instrument and data quality

A well-structured, self-developed, and validated questionnaire was developed based on a previous literature review [12, 14, 16] and was used as the primary data-collection tool for gathering information. The dependent variable was Spinal Induced Anesthesia Hypotension (SIAH). The independent variables were divided into three parts: the first part was sociodemographic (age, BMI, pregnancy type, surgery type, and gravidity); the second part was medical history (previous cesarean section, history of hypertension in pregnancy, baseline systolic blood pressure, baseline heart rate, fasting duration); and the third part was anesthetic characteristics (preload IV fluid, amount of preload IV fluid, time duration IV preloaded giving, sitting position five minutes after intrathecal injection, level of spinal block, metoclopramide medication, ephedrine medication, and phenylephrine medication). This study was approved by the Ethics Committee Board of Mogadishu Somali Turkey Training and Research Hospital. Pregnant women who met the inclusion criteria were enrolled in the study. Written informed consent was obtained from every participant. Caesarean sections were performed under spinal anesthesia performed by physician anesthetists. The questionnaires were completed through interviews and medical records.

For the questionnaire, the item objective congruence (IOC) method [17] was used to improve content validity by three external experts: two anesthesiologists and one obstetrician working in the field. Experts were selected based on their experience in the clinical work of pregnant patients under spinal anesthesia. Each expert provided a score for each item with comments:1 for clearly relevant to the study, -1 for clearly not relevant to the study, and 0 for the content area was unclear regarding connection with the study. For the results, any question that scored ≤ 0.5 was deleted from the questionnaire, any question that scored 0.5—0.7 was revised according to the comments, and any question that scored > 0.7 was included in the questionnaire.

For the reliability test, the questionnaire was developed in English and backward forward translation was conducted by fluent experts in both languages. Before data collection, a pilot test was conducted at the study hospital, with 30 patients sharing similar characteristics with the study sample. The reliability, feasibility, and order of the questions were determined. This test assessed the reliability, feasibility, and question order. The questionnaire demonstrated acceptable reliability, with a Cronbach’s alpha of 0.78.

### Operational definitions

*Spinal induced anesthesia hypotension (SIAH)* is defined as a reduction in systolic blood pressure from baseline values or a systolic blood pressure of less than 90 mmHg at any point after the initiation of spinal anesthesia [18].

*Cesarean section* is a surgical procedure in which a baby is born through a cut made in the mother’s abdominal wall and uterus [19].

*Spinal anesthesia* is a type of regional anesthesia in which a local anesthetic is injected directly into the cerebrospinal fluid surrounding the spinal cord and nerve roots [20].

### Data analysis process

Data were double-entered into an Excel spreadsheet and checked for errors before being transferred into SPSS version 20.0 (Chicago, IL, USA) for analysis. Descriptive statistics were used to present the frequencies and percentages of the categorical data. The chi-square test was used to compare patients with hypotension who underwent cesarean section under spinal anesthesia and those who did not. Logistic regression was used to identify factors associated with hypotension in patients who underwent cesarean section after spinal anesthesia. Variables that were significant at P-value of < 0.20 level by univariate analysis were included and retained in the multivariate model, as recommended by Bursac et al., to detect all significant risk factors while controlling for adjusting all possible confounders [21]. The final model presented adjusted odds ratios (AOR) with 95% confidence intervals (CI) at a significance level of α = 0.05. The Hosmer-Lemeshow goodness-of-fit test was used to evaluate the assumptions of good predictable variables in multivariate regression at *p* > 0.05 [22]. All findings are presented in the tables and text.

## Results

A total of 320 participants were included in this analysis, and the incidence of SIAH was 78.1%. 3.5% were mothers aged between 21 and 35 years, and 50.3% had a BMI above 25 kg/m^2^ (Table 1).

61.6% of the participants had no previous experience of cesarean Sect. (61.6%), 89.1% had no history of hypertension in pregnancy, and 88.1% had baseline systolic blood pressure less than or equal to 120mmHg (Table 2).

83.8% had normal range of blood pressure of prior induction of anesthesia, 86.6% had no intravenous fluid preload, 64.1% had SAB administered at L4/L5, 84.7% had no medication with metoclopramide, 53.4% had no ephedrine medication, 50.3% were between 1000 and 1500 ml of intraoperative crystalloid, 59.7% were above 11 g/dL preoperative hemoglobin, and 32.8% had non-reassuring fetal status for indication of c/s (Table 3).

Among sociodemographic characteristics, age, pregnancy type, surgery type, and gravidity were not significantly associated with hypotension in C/S patients after SA, whereas BMI (kg/M^2^) was significantly associated with hypotension in C/S patients after SA (*p* = 0.026) (Table 1).

Among the medical history characteristics, fasting duration and baseline heart rate (bpm) were not significantly associated with hypotension in C/S patients after SA, whereas previous cesarean section (*p* = 0.028), history of hypertension during pregnancy (*p* < 0.001), and baseline systolic blood pressure (*p* < 0.001) were significantly associated with hypotension in C/S patients after SA (Table 2).

Among the anesthetic characteristics, preload IV Fluid, duration IV preloading given minutes, patient positioning after spinal, phenylephrine medication, time interval in minutes between spinal induction, and skin incision were not significantly associated with hypotension in C/S patients after SA, while prior anesthesia blood pressure (*p* ≤ 0.001), level of spinal block (*p* = 0.013), metoclopramide medication (*p* = 0.047), ephedrine medication (*p* < 0.001), amount of millimeter crystalloid intraoperatively (*p* = 0.006), preoperative hemoglobin (*p* < 0.001), and indication for C/S (*p* < 0.001) were significantly associated with hypotension in C/S patients after SA (Table 3).

**Table 1:** Social demographic characteristics and comparison between dependent and independent variables, n (320) 2024

Variables	Total *n* (%)	SIAH		*P*-value
		Yes *n* (%)	No *n* (%)	
Total	320 (100%)	250 (78.1)	70 (21.9)	
*Age (Years)*				
Less than or equal 20	35 (10.9)	23 (9.2)	12 (17.1)	0.083
21–35	235 (73.5)	184 (73.6)	51 (72.9)	
> 35	50 (15.6)	43 (17.2)	7 (10.0)	
*BMI (Kg/M* ^*2*^ *)*				
Less than or equal 25	159 (49.7)	116 (46.4)	43 (61.4)	**0.026**
> 25	161 (50.3)	134 (53.6)	27 (38.6)	
*Pregnancy type*				
Singleton	303 (94.7)	239 (95.6)	64 (91.4)	0.223^a^
Twins	17 (5.3)	11 (4.4)	6 (8.6)	
*Surgery type*				
Emergency	194 (60.6)	148 (59.2)	46 (65.7)	0.324
Elective	126 (39.4)	102 (40.8)	24 (34.3)	
*Gravidity*				
1	59 (18.4)	46 (18.4)	13 (18.6)	0.196
2	92 (28.8)	65 (26.0)	27 (38.6)	
3–4	113 (35.3)	93 (37.2)	20 (28.6)	
Greater than or equal 5	56 (17.5)	46 (18.4)	10 (14.3)	

The p-value (bold) was calculated using the chi-square test to assess the association between the dependent and independent variables, with a significance level of α = 0.05

^a^Fisher’s exact test

**Table 2:** Medical history characteristics and comparison between dependent and independent variables, n (320) 2024

Variables	Total *n* (%)	SIAH		*P*-value
		Yes *n* (%)	No *n* (%)	
*Previous cesarean section*				
Yes	123 (38.4)	104 (41.6)	19 (27.1)	**0.028**
No	197 (61.6)	146 (58.4)	51 (72.9)	
*History hypertension in pregnancy*				
Yes	35 (10.9)	16 (6.4)	19 (27.1)	**< 0.001**
No	285 (89.1)	234(93.6)	51 (72.9)	
*Baseline systolic blood pressure*				
Less than or equal 120	282 (88.1)	231 (92.4)	51 (72.9)	**< 0.001**
> 120	38 (11.9)	19 (7.6)	19 (27.1)	
*Baseline heart rate (bpm)*				
Less than or equal 100	299 (93.4)	235 (94.0)	64 (91.4)	0.421^a^
> 100	21 (6.6)	15 (6.0)	6 (8.6)	
*Fasting duration (hours)*				
Yes	242 (75.6)	187 (74.8)	55 (78.6)	0.516
No	78 (24.4)	63 (25.2)	15 (21.4)	

The p-value (bold) was calculated using the chi-square test to assess the association between the dependent and independent variables, with a significance level of α = 0.05

^a^Fisher’s exact test

**Table 3:** Anesthetics characteristics and comparison between dependent and independent variables, n (320) 2024

Variables	Total *n* (%)	SIAH		*P*-value
		Yes *n* (%)	No *n* (%)	
*BP prior induction*				
Normal Bp	268 (83.8)	219 (87.6)	49 (70.0)	**< 0.001**
Low Bp	15 (4.7)	13(5.2)	2 (2.9)	
High Bp	37 (11.6)	18(7.2)	19 (27.1)	
*Preload*				
Yes	43 (13.4)	29 (11.6)	14 (20.0)	0.069
No	277 (86.6)	221 (88.4)	56 (80.0)	
*Volume of preload (ml)*				
Less than or equal 500	24 (7.5)	15 (51.7)	9 (64.3)	0.437
> 500	19 (5.9)	5(35.7)	14 (48.3)	
*Duration of preload (min)*				
Less than or equal 5	14(4.4)	9 (3.6)	5 (7.1)	0.434
> 5	22(6.9)	17 (6.8)	5 (7.1)	
None	284(88.7)	224 (89.6)	60(85.7)	
*Patient positioning after spinal*				
Leg wrapping	7(2.2)	5 (2.0)	2 (2.9)	0.643^a^
Left lateral table tilt	10(3.1)	9 (3.6)	1 (1.4)	
None	303(94.7)	236 (94.4)	67 (95.7)	
*Interspace at which SAB was administered*				
L3-L4	115(35.9)	81 (32.4)	34 (48.6)	**0.013**
L4-L5	205(64.1)	169 (67.6)	36 (51.4)	
*Metoclopramide medication*				
Yes	49(15.3)	33 (13.2)	16 (22.9)	**0.047**
No	271(84.7)	217 (86.8)	54 (77.1)	
*Ephedrine medication*				
Yes	149 (46.6)	93 (37.2)	56 (80.0)	**< 0.001**
No	171 (53.4)	157 (62.8)	14 (20.0)	
*Phenylephrine medication*				
Yes	58 (18.1)	45 (18.0)	13 (18.6)	0.913
No	262 (81.9)	205 (82.0)	57 (81.4)	
*Interval between spinal induction and skin incision (min)*				
5–6	301(94.1)	233 (93.2)	68 (97.1)	0.267
> 6	19 (5.9)	17 (6.8)	2 (2.9)	
*Amount of crystalloid intraoperative (ML)*				
1000	37(11.6)	22 (8.8)	15 (21.4)	**0.006**
1000	161(50.3)	125 (50.0)	36 (51.4)	
> 1500	122(38.1)	103 (41.2)	19 (27.1)	
*Preoperative hemoglobin*				
Above 11 g/dl	191(59.7)	136 (54.4)	55 (78.6)	**< 0.001**
Below 11 g/dl	129(40.3)	114 (45.6)	15 (21.4)	
*Indication for C/S*				
Malpresentation	84(26.2)	69 (27.6)	15 (21.4)	**< 0.001**
Non-reassuring fetal status	105(32.8)	81 (32.4)	24 (34.3)	
Previous scar	94(29.4)	82 (32.8)	12 (17.1)	
Uterine rapture	8(2.5)	6 (2.4)	2 (2.9)	
Pregnancy-related high blood pressure disorders	29 (9.1)	12 (4.8)	17 (24.3)	

The p-value (bold) was calculated using the chi-square test to assess the association between the dependent and independent variables, with a significance level of α = 0.05

^a^Fisher’s exact test

### Univariate analysis of factors associated with spinal induced anesthesia hypotension

Table 4 shows the results of the univariate associations between the independent variables and patients with spinal induced hypotension undergoing C/S. These variables crude odds ratios less than 0.20 including: age (years), BMI (Kg/M^2^), pregnancy type, previous cesarean section, history of hypertension in pregnancy, baseline systolic blood pressure, prior anesthesia blood pressure, preload IV Fluid, level of spinal block, metoclopramide medication, ephedrine medication, amount of crystalloid intraoperatively (ML), preoperative hemoglobin level, and indication for c/s were significantly associated with hypotension in c/s patients after SA.

### Multivariate analyses of factors associated with spinal induced anesthesia hypotension

Table 4 shows the results of the multivariate logistic regression model for factors associated with hypotension in patients with c/s after SA. Only five (5) variables were found to be associated with hypotension in c/s patients after SA: Participants with BMI > 25 kg/m^2^ had 2.65 times (95%CI = 1.39–5.04) greater odds of developing hypotension in c/s patients after SA than those with BMI ≤ 25. Participants with no history of hypertension during pregnancy had a 6.00 times (95%CI = 2.50–14.40) greater risk of having hypotension in c/s patients after SA than those with a history of hypertension in pregnancy. Participants who did not take preload iv fluid had 3.06 times (95%CI = 1.22–7.66) greater risk of having hypotension C/S patients after SA than those who took preload iv fluid. Participants who did not take ephedrine medication had 8.84 times (95%CI = 4.10-19.05) greater risk of having hypotension C/S patients after SA than those who took ephedrine medication. Participants with < 11 g/dl preoperative hemoglobin had 2.52 times (95%CI = 1.25–5.05) more likely to have hypotension in c/s patients after SA than those > 11 g/dl with preoperative hemoglobin.

**Table 4:** Factors associated with hypotension after spinal anesthesia in univariable and multivariable analyse among C/S patients admitted to the hospital in mogadishu, Somalia 2024, (*n* = 320)

Variables	OR (95%CI)	*P*-value	AOR (95%CI)	*P*-value
*Age (Years)*				
Less than or equal 20	1			
21–35	1.88 (0.88–4.04)	0.105		
> 35	3.21 (1.11–9.26)	**0.031**		
*BMI (Kg/M* ^*2*^ *)*				
Less than or equal 25	1		1	
> 25	1.84 (1.07–3.16)	**0.027**	2.65 (1.39–5.04)	**0.003**
*Pregnancy type*				
Singleton	1			
Twins	0.49 (0.18–1.38)	0.177		
*Surgery type*				
Emergency	0.76 (0.44–1.32)	0.325		
Elective	1			
*Gravidity*				
1	1			
2	0.68 (0.32–1.46)	0.322		
3–4	1.31 (0.60–2.87)	0.494		
Greater than or equal 5	1.30 (0.52–3.26)	0.576		
*Previous cesarean section*				
Yes	1.91 (1.07–3.42)	**0.030**		
No	1			
*History hypertension in pregnancy*				
Yes	1		1	
No	5.45 (2.62–11.32)	**< 0.001**	6.00 (2.50–14.40)	**< 0.001**
*Baseline systolic blood pressure*				
Less than or equal 120	4.53 (2.24–9.16)	**< 0.001**		
> 120	1			
*Baseline heart rate (bpm)*				
Less than or equal 100	1			
> 100	0.69 (0.25–1.83)	0.445		
*Fasting duration (hours)*				
Yes	1			
No	1.24 (0.65–2.34)1	0.516		
*BP prior induction*				
Normal Bp	1			
Low Bp	1.45 (0.32–6.65)	0.629		
High Bp	0.21 (0.10–0.43)	**< 0.001**		
*Preload*				
Yes	1		1	
No	1.91 (0.94–3.84)	0.072	3.06 (1.22–7.66)	**0.017**
*Volume of preload (ml)*				
Less than or equal 500	0.60 (0.16–2.21)	0.439		
> 500	1			
*Duration of preload (min)*				
Less than or equal 5	1.89 (0.43–8.30)	0.400		
> 5	1			
None	2.07 (0.67–6.42)	0.206		
*Patient positioning after spinal*				
Leg wrapping	0.71 (0.14–3.74)	0.686		
Left lateral table tilt	2.56 (0.32–20.53)	0.378		
None	1			
*Interspace at which SAB was administered*				
L3-L4	1			
L4-L5	1.97 (1.15–3.38)	**0.014**		
*Metoclopramide medication*				
Yes	1			
No	1.95 (1.00-3.80)	**0.050**		
Ephedrine medication				
Yes	1		1	
No	6.75 (3.56–12.80)	**< 0.001**	8.84 (4.10-19.05)	**< 0.001**
*Phenylephrine medication*				
Yes	1			
No	1.04 (0.53–2.06)	0.913		
*Interval between spinal induction and skin incision (min)*				
5–6	1			
> 6	2.48 (0.56–11.01)	0.232		
*Amount of crystalloid intraoperative (ML)*				
1000	3.70 (1.63–8.38)	**0.002**		
1000–1500	2.37 (1.11–5.03)	**0.025**		
> 1500	1			
*Preoperative hemoglobin*				
Above 11 g/dl	1		1	
Below 11 g/dl	3.07 (1.65–5.73)	**< 0.001**	2.52 (1.25–5.05)	**0.010**
*Indication for C/S*				
Malpresentation	1			
Non-reassuring fetal status	0.73 (0.36–1.51)	0.400		
Previous scar	1.49 (0.65–3.39)	0.346		
Uterine rapture	0.65 (0.12–3.55)	0.621		
Pregnancy-related high blood pressure disorders	0.15 (0.06–0.39)	**< 0.001**		

1 = Reference

The significance (bold) level was set at α = 0.05

## Discussion

This study found a high incidence, which was higher than that reported in studies conducted in Pakistan [23], Ethiopia [16, 24], and Columbia [25]. These differences suggest potential variability in local risk factors, healthcare access, and lifestyle differences, which may influence the incidence rates across these regions. Notably, this study offers important insights into the determinants of spinal anesthesia-induced hypotension in this context, emphasizing the need for improved management strategies. These findings may guide the development of targeted interventions and evidence-based guidelines to reduce the incidence of spinal anesthesia-induced hypotension, thereby improving patient safety and advancing maternal and neonatal care in low-resource settings.

According to the study’s final model, hypotension in patients undergoing cesarean section under spinal anesthesia induced hypotension (SAIH) was significantly related to five variables. Interestingly, mothers with BMI > 25 kg/m^2^ had greater odds of developing hypotension c/s after SA. This finding is supported by a study conducted in Iran [12]. A possible explanation for this finding, particularly in the Somali context, is that mothers with a BMI above 25 kg/m² may be more susceptible to hypotension due to increased intra-abdominal pressure on major blood vessels, reduced cardiovascular reserve, and limited access to specialized care. These factors may hinder optimal management of obesity-related complications, including accurate anesthetic dosing and timely intervention for blood pressure fluctuations. These challenges can increase risks in resource-limited settings. A potential mechanism for the more common spinal blockage in these patients appears to be a reduction in CSF volume caused by increased abdominal pressure and compression of the subarachnoid space, as in obesity or pregnancy [12]. Anesthesiologists closely monitor and administer vasopressors more frequently to patients with a higher BMI to counteract these risks during cesarean section under spinal anesthesia.

A notable finding of this study is that mothers without a history of hypertension during pregnancy were more likely to experience hypotension following spinal anesthesia. Although unexpected, this aligns with evidence from Ethiopia showing lower hypotension rates among preeclamptic women [26]. A likely explanation is that normotensive women have lower vascular resistance and sympathetic tone, which reduces their ability to compensate for spinal anesthesia-induced vasodilation. In contrast, hypertensive or preeclamptic patients may benefit from higher baseline vascular tone. This highlights the need for vigilant intraoperative monitoring and proactive management, even in patients without known risk factors. Further research is recommended to confirm this association and understand the underlying mechanisms.

One of the key findings of this study was that mothers who did not receive preload IV fluids before spinal anesthesia were significantly more likely to develop hypotension during cesarean section. This association can be attributed to the physiological effects of spinal anesthesia, which induces sympathetic blockade, resulting in peripheral vasodilation, venous pooling in the lower extremities, and a subsequent reduction in venous return and cardiac output—culminating in a decline in blood pressure. Preloading with IV fluids serves to counteract these hemodynamic changes by augmenting intravascular volume, thereby supporting cardiac output and mitigating the severity of hypotension. In low-resource settings such as Somalia, where access to advanced hemodynamic monitoring and pharmacologic interventions may be limited, fluid preloading represents a crucial and practical preventive measure. This finding aligns that in the existing literature. For instance, a study conducted in Iraq reported that preloading significantly reduced the incidence of spinal anesthesia-induced hypotension among women undergoing cesarean Sect. [27]. Similarly, evidence from Iran demonstrated that administration of higher volumes of preload fluid was associated with a significantly lower occurrence of maternal hypotension compared to smaller volumes [12].

Another important finding was that mothers who did not receive ephedrine medication had a greater risk of hypotension after spinal anesthesia (SA) for cesarean section (C/S) owing to the physiological effects of SA. This might be a possible explanation because spinal anesthesia causes significant vasodilation and reduced blood pressure, which are more pronounced in patients without interventions such as ephedrine. Ephedrine is a vital preventive measure in resource-constrained settings as it improves vascular tone and cardiac output. Therefore, healthcare facilities in Somalia face challenges such as inadequate monitoring equipment and limited availability of alternative medications, making ephedrine more critical. Research in India has discovered that ephedrine is both safe and effective in preventing and treating hypotension during spinal anesthesia, although it is more effective than other sympathomimetic drugs [28]. Studies conducted in India [28] and Pakistan [29] support this finding.

Finally, this study found that mothers with preoperative hemoglobin levels below 11 g/dL were significantly more likely to experience hypotension after spinal anesthesia. This may be due to the reduced oxygen-carrying capacity and limited compensatory cardiovascular response in patients with anemic undergoing sympathetic blockade. Interestingly, this contrasts with a study conducted in Kenya, which identified higher hemoglobin levels as a risk factor for hypotension [30]. Such discrepancies may be attributed to variations in study design, population characteristics, or confounding variables. Nonetheless, the present finding suggests that optimizing maternal hemoglobin levels prior to surgery may reduce the risk of hypotension, particularly in low-resource settings.

## Conclusion

This study identified a high incidence of spinal anesthesia-induced hypotension at a referral hospital in Mogadishu, Somalia. Key risk factors included a body mass index above 25 kg/m², absence of hypertensive disorders during pregnancy, failure to administer preload intravenous fluids, lack of vasopressor use (particularly ephedrine), and low preoperative hemoglobin levels below 11 g/dL. Patients with anemia and those who did not receive vasopressors were particularly vulnerable. Based on these findings, the study recommends early screening and treatment of maternal anemia during antenatal care, assessment and preparation for overweight patients, and routine administration of preloaded IV fluids, especially in normotensive mothers. It also emphasizes the importance of incorporating vasopressors like ephedrine into perioperative protocols for high-risk patients, enhancing anesthesia provider training, and developing local evidence-based guidelines for managing maternal hypotension. Furthermore, addressing these modifiable risks through targeted strategies could significantly reduce the incidence of spinal anesthesia-induced hypotension and improve maternal outcomes.

### Strength and limitation of the study

The strength of this study is that it provides valuable incidence data and identifies key risk factors for hypotension in cesarean section patients under spinal anesthesia at a referral hospital in Mogadishu, Somalia. However, they also offer important contributions to local health care practices. The main limitation of this study is its cross-sectional design, which limits the ability to infer causal relationships between the independent variables and hypotension. Additionally, the lack of consistent documentation on anesthetic dosage and spinal block height—due to reliance on routine medical records—precluded the analysis of their effects. Future larger, prospective studies should investigate these factors to better understand the mechanisms of spinal anesthesia-induced hypotension (SAIH).

## Data Availability

All the data are presented in this manuscript. Additional data can be obtained from the corresponding author upon request.

## Abbreviations

SAIH: Spinal anesthesia-induced hypotension
SA: Spinal anesthesia
C/S: Cesarean section
CSF: Cerebrospinal fluid
BMI: Body mass index
BP: Blood pressure
IV: Intravenous
ML: Milliliter
bpm: Beats per minute
SAB: Subarachnoid block
